# Supplementary material for: Optogenetic control shows that kinetic proofreading regulates the activity of the T cell receptor
Source: eLife. 2019 Apr 5;8:e42475. doi: 10.7554/eLife.42475 (PMC6488296; doi:10.7554/eLife.42475)
Supplement: Supplementary file 1. — Plasmids generated in this study were listed next to the protein each plasmid codes for and a brief description. Detailed descriptions of the cloning strategies are available upon request. [file elife-42475-supp1.docx]

| **Plasmid** | **Expressed protein** | **Description** | **Cloning strategy** |
| --- | --- | --- | --- |
| pOSY015 | PIF(wt)-TCRβ | PIF6_1-100_ from pHB002 ([Beyer et al., 2018](#_ENREF_3)) fused to HA1.7 TCRβ ([Hennecke et al., 2000](#_ENREF_11); [Hewitt et al., 1992](#_ENREF_12)) inside lentiviral expression plasmid pLVX (Clontech) | Ligation of pOSY002 (unpublished) EcoRI-FseI fragment with pOSY012 (unpublished) FseI-EcoRI fragment |
| pOSY016 | PIF(Q)-TCRβ | PIF6_1-100_ N35Q mutation in pOSY015 | Ligation of pOSY004 (unpublished) EcoRI-FseI fragment with pOSY012 (unpublished) FseI-EcoRI fragment |
| pOSY017 | PIF(A)-TCRβ | PIF6_1-100_ S37A mutation in pOSY015 | Ligation of pOSY006 (unpublished) EcoRI-FseI fragment with pOSY012 (unpublished) FseI-EcoRI fragment |
| pOSY019 | scFv-TCRβ | scFv fused to HA1.7 TCRβ ([Minguet et al., 2007](#_ENREF_18)) inside lentiviral expression plasmid pLVX (Clontech) | Ligation of pOSY008 (unpublished) EcoRI-FseI fragment with pOSY012 (unpublished) FseI-EcoRI fragment |
| pOSY026 | PIF(SS)-TCRβ | PIF6_1-100_ C9S and C10S mutation in pOSY015 | Mutagenesis PCR with O058 fw and O059 rv from pOSY015 |
| pOSY027 | PIF(SSQ)-TCRβ | PIF6_1-100_ C9S and C10S mutation in pOSY016 | Mutagenesis PCR with O058 fw and O059 rv from pOSY016 |
| pOSY028 | PIF(SSA)-TCRβ  = PIF^S^-TCRβ | PIF6_1-100_ C9S and C10S mutation in pOSY017 | Mutagenesis PCR with O058 fw and O059 rv from pOSY017 |
| pOSY061 | MBP-PIF(wt) | MBP fused to PIF6_1-100_ inside bacterial expression vector pMAL-c5x (New England Biolabs) | Gibson of two fragments: 1) EcoRI-SacI fragment of pMH05 (unpublished); 2) PCR with O152 fw and O154 rv from PCR with O151 fw and O153 rv from pOSY015 |
| pOSY062 | MBP-PIF(Q) | PIF6_1-100_ N35Q mutation in pOSY061 | Gibson of two fragments: 1) EcoRI-SacI fragment of pMH05 (unpublished); 2) PCR with O152 fw and O154 rv from PCR with O151 fw and O153 rv from pOSY016 |
| pOSY063 | MBP-PIF(A) | PIF6_1-100_ S37A mutation in pOSY063 | Gibson of two fragments: 1) EcoRI-SacI fragment of pMH05 (unpublished); 2) PCR with O152 fw and O154 rv from PCR with O151 fw and O153 rv from pOSY017 |
| pOSY064 | MBP-PIF(SS) | PIF6_1-100_ C9S and C10S mutation in pOSY061 | Gibson of two fragments: 1) EcoRI-SacI fragment of pMH05 (unpublished); 2) PCR with O152 fw and O154 rv from PCR with O155 fw and O153 rv from pOSY026 |
| pOSY065 | MBP-PIF(SSQ) | PIF6_1-100_ C9S, C10S and N35Q mutation in pOSY061 | Gibson of two fragments: 1) EcoRI-SacI fragment of pMH05 (unpublished); 2) PCR with O152 fw and O154 rv from PCR with O155 fw and O153 rv from pOSY027 |
| pOSY066 | MBP-PIF(SSA) | PIF6_1-100_ C9S, C10S and S37A mutation in pOSY061 | Gibson of two fragments: 1) EcoRI-SacI fragment of pMH05 (unpublished); 2) PCR with O152 fw and O154 rv from PCR with O155 fw and O153 rv from pOSY028 |
| pOSY073 | GFP-F1-PIF^S^-TCRβ | moxGFP ([Costantini et al., 2015](#_ENREF_5)) followed by furin site F1 (GSRRKRSVSG) fused in between signal peptide and PIF^S^ inside pOSY028 | Gibson of two fragments: 1) PspXI-SpeI fragment of pOSY028; 2) PCR with O169 fw and O174 rv from PCR with O168 fw and O170 rv from moxGFP (Erik Snapp, Addgene plasmid #68070) |
| pOSY074 | GFP-F2-PIF^S^-TCRβ | Furin site F2 (GIRRKRSVSH) instead of F1 inside pOSY073 | Gibson of two fragments: 1) PspXI-SpeI fragment of pOSY028; 2) PCR with O169 fw and O174 rv from PCR with O168 fw and O171 rv from moxGFP (Erik Snapp, Addgene plasmid #68070) |
| pOSY075 | GFP-F3-PIF^S^-TCRβ | Furin site F3 (GSRRKR) instead of F1 inside pOSY073 | Gibson of two fragments: 1) PspXI-SpeI fragment of pOSY028; 2) PCR with O169 fw and O174 rv from PCR with O168 fw and O172 rv from moxGFP (Erik Snapp, Addgene plasmid #68070) |
| pOSY076 | GFP-noF-PIF^S^-TCRβ | Flexible linker noF (GGSG) instead of F1 inside pOSY073 | Gibson of two fragments: 1) PspXI-SpeI fragment of pOSY028; 2) PCR with O169 fw and O174 rv from PCR with O168 fw and O173 rv from moxGFP (Erik Snapp, Addgene plasmid #68070) |
