# Supplementary material for: Optogenetic control shows that kinetic proofreading regulates the activity of the T cell receptor
Source: eLife. 2019 Apr 5;8:e42475. doi: 10.7554/eLife.42475 (PMC6488296; doi:10.7554/eLife.42475)
Supplement: Supplementary file 2. [file elife-42475-supp2.docx]

| **Primer** | **Sequence** |
| --- | --- |
| O058 | ACTCCATGATGTTCTTACCAACCGATTACTCGAGCAGGTTAAGCGATCAAGAG |
| O059 | CTCTTGATCGCTTAACCTGCTCGAGTAATCGGTTGGTAAGAACATCATGGAGT |
| O151 | CTGGAAGTGCTGTTTCAGGGCCCGCTCGACGGTTCAGGTGGAGATGTGATGATGTTCTTACCAACCGATTATTGTTG |
| O152 | GCCCTGAAAGACGCGCAGACTAATTCGAGCTCGAACAACAACAACAATAACAATAACAACAACCTCGGGCTGGAAGTGCTGTTTCAGGG |
| O153 | TTAGTGATGGTGATGATGGTGGTCAACATGTTTATTGCTTTCCA |
| O154 | TATTTGAAGCTTATTTAATTACCTGCAGGGAATTCTTAGTGATGGTGATGATGGTGG |
| O155 | CTGGAAGTGCTGTTTCAGGGCCCGCTCGACGGTTCAGGTGGAGATGTGATGATGTTCTTACCAACCGATTACTC |
| O168 | ATGGGATGGAGCTGTATCATGCTCTTCTTGGCAGCAACAGCTACAGGTGTCCACTCCTCCAAGGGCGAGGAGCTG |
| O169 | CCGGATCTATTTCCGGTGAATTCCTCGAGACTAGTGCCACCATGGGATGGAGCTGTATCATG |
| O170 | GTAATCGGTTGGTAAGAACATCATTCCAGACACGGATCTCTTTCTCCTGCTGCCCTTGTACAGCTCGTCCATGCC |
| O171 | GTAATCGGTTGGTAAGAACATCATGTGTGAGACGCTTCGTTTACGTCTTATACCCTTGTACAGCTCGTCCATGCC |
| O172 | GTAATCGGTTGGTAAGAACATCATTCTCTTTCTCCTGCTGCCCTTGTACAGCTCGTCCATGCC |
| O173 | GTAATCGGTTGGTAAGAACATCATGCCAGAACCTCCCTTGTACAGCTCGTCCATGCC |
| O174 | CATATACTCTTGATCGCTTAACCTGCTCGAGTAATCGGTTGGTAAGAACATCAT |
