# Supplementary figures and images for: Optogenetic control shows that kinetic proofreading regulates the activity of the T cell receptor
Source: eLife. 2019 Apr 5;8:e42475. doi: 10.7554/eLife.42475 (PMC6488296; doi:10.7554/eLife.42475)

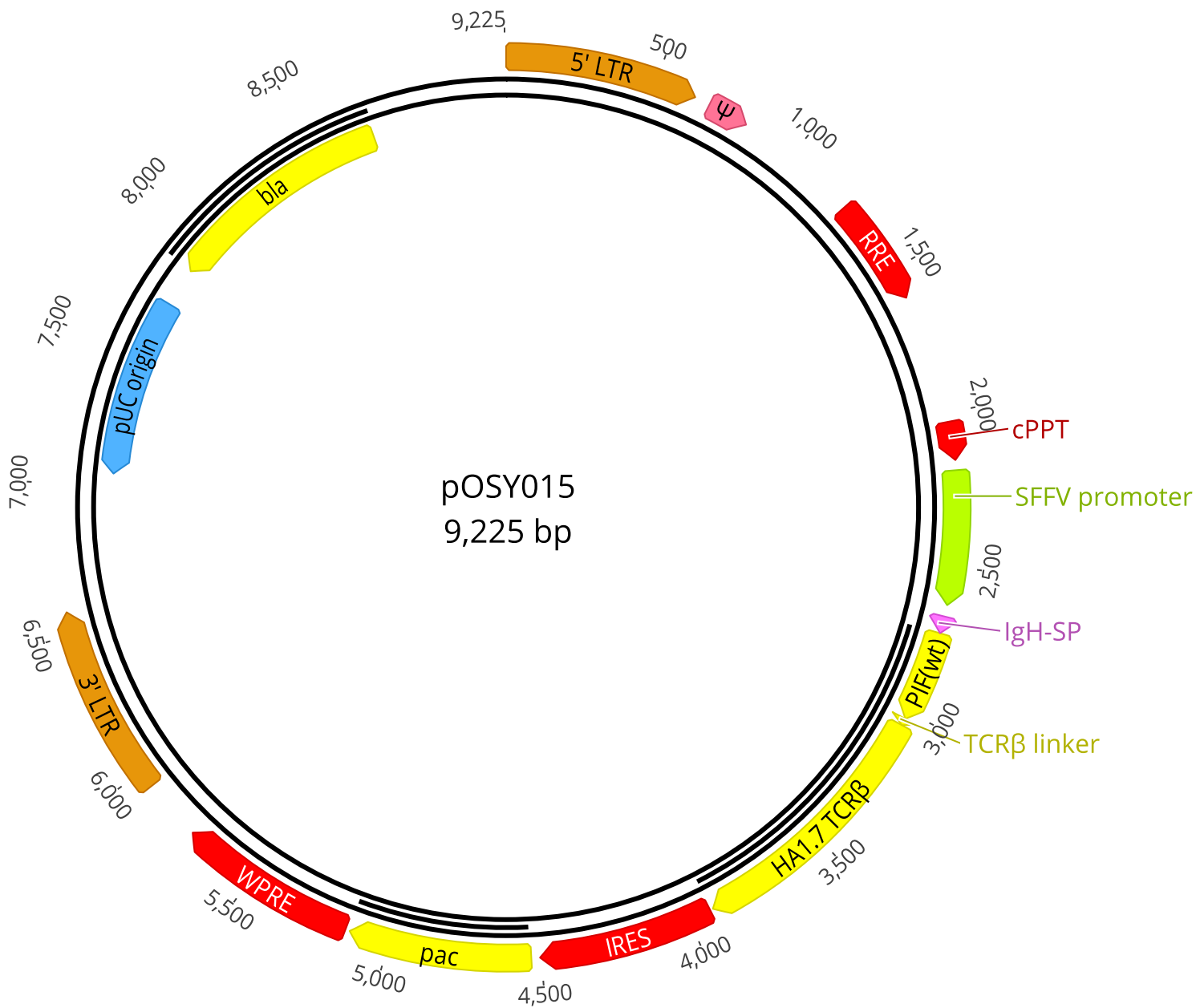

Supplement: Supplementary file 3. [file elife-42475-supp3.pdf]

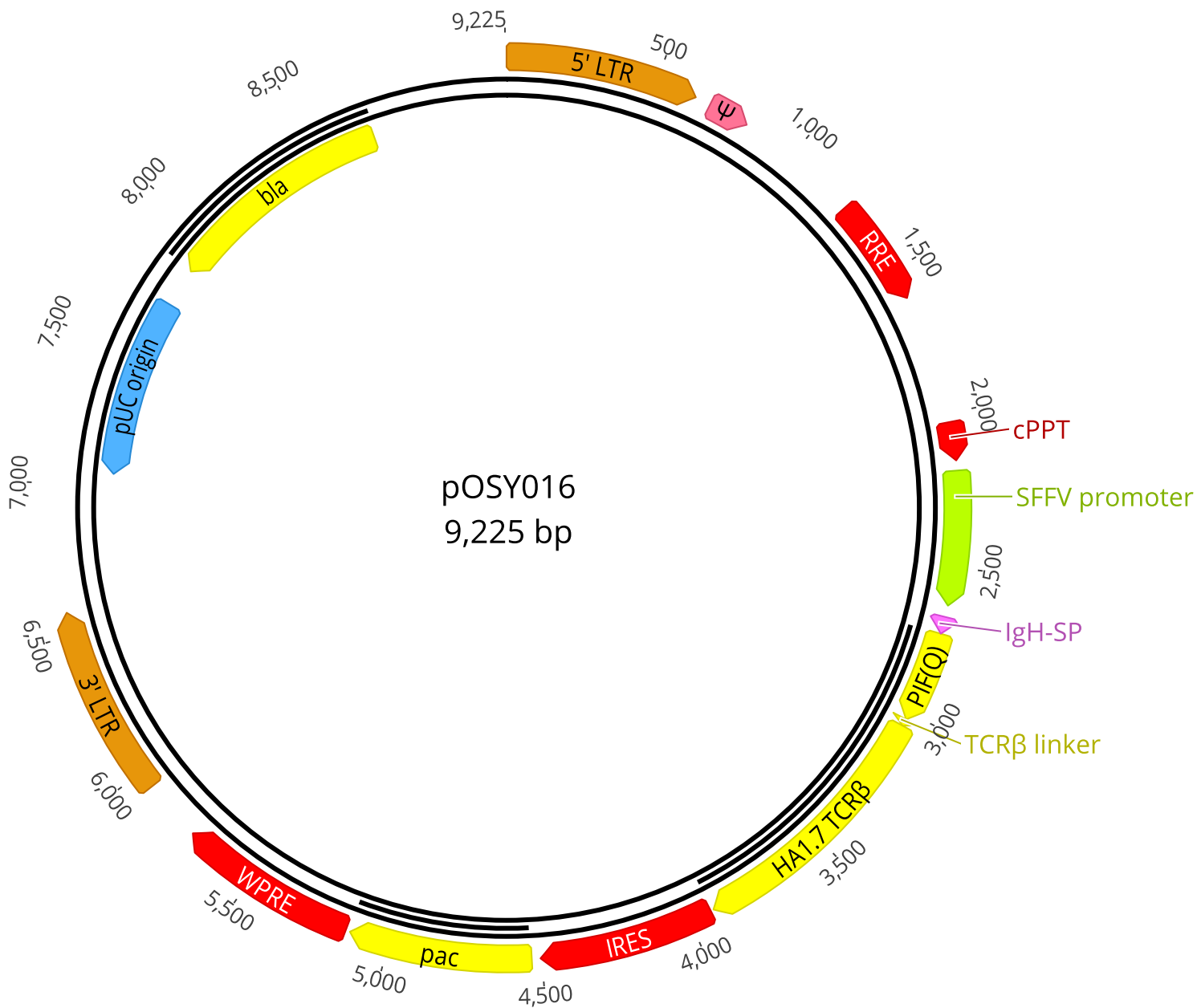

Supplement: Supplementary file 6. [file elife-42475-supp6.pdf]

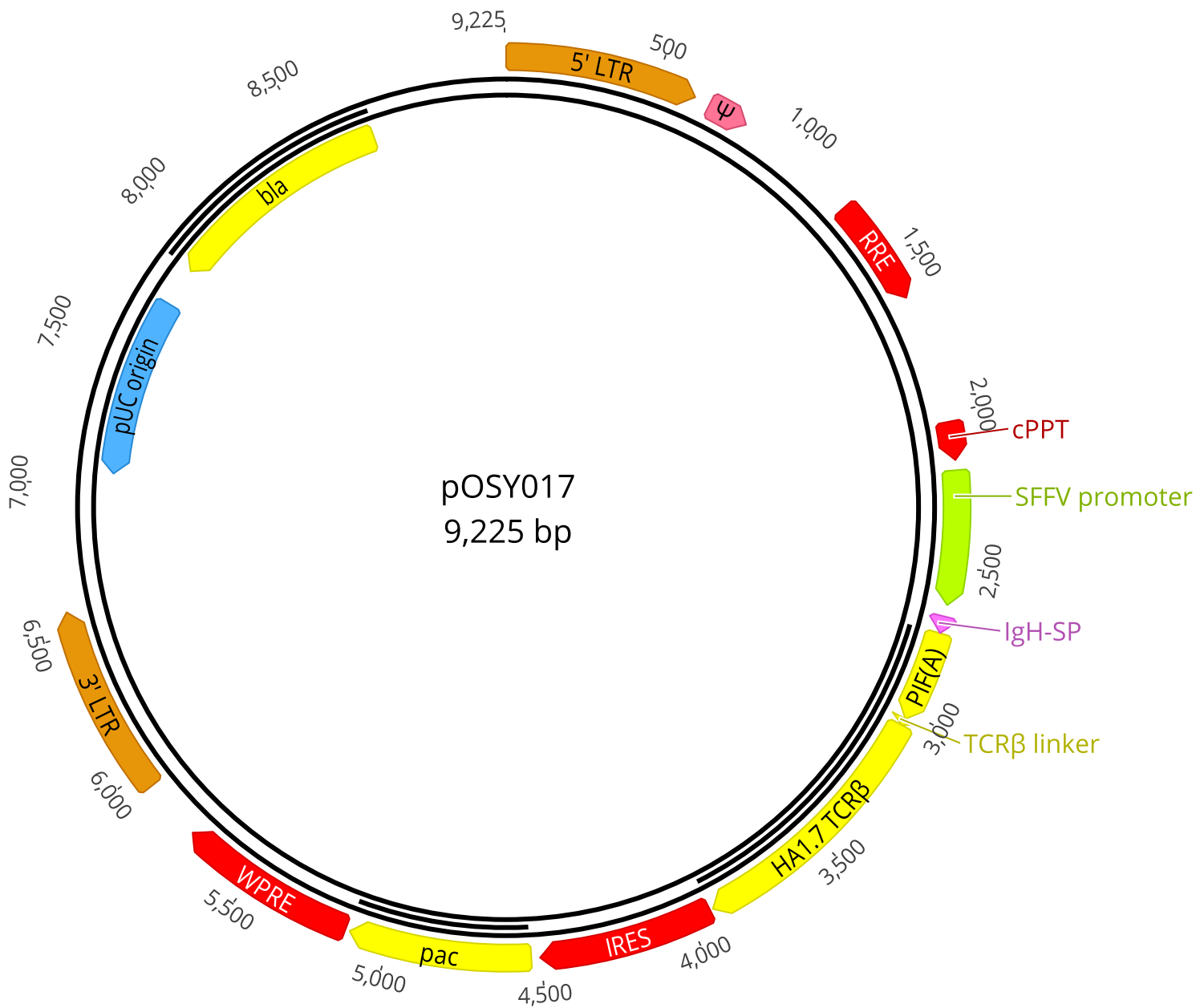

Supplement: Supplementary file 8. [file elife-42475-supp8.pdf]

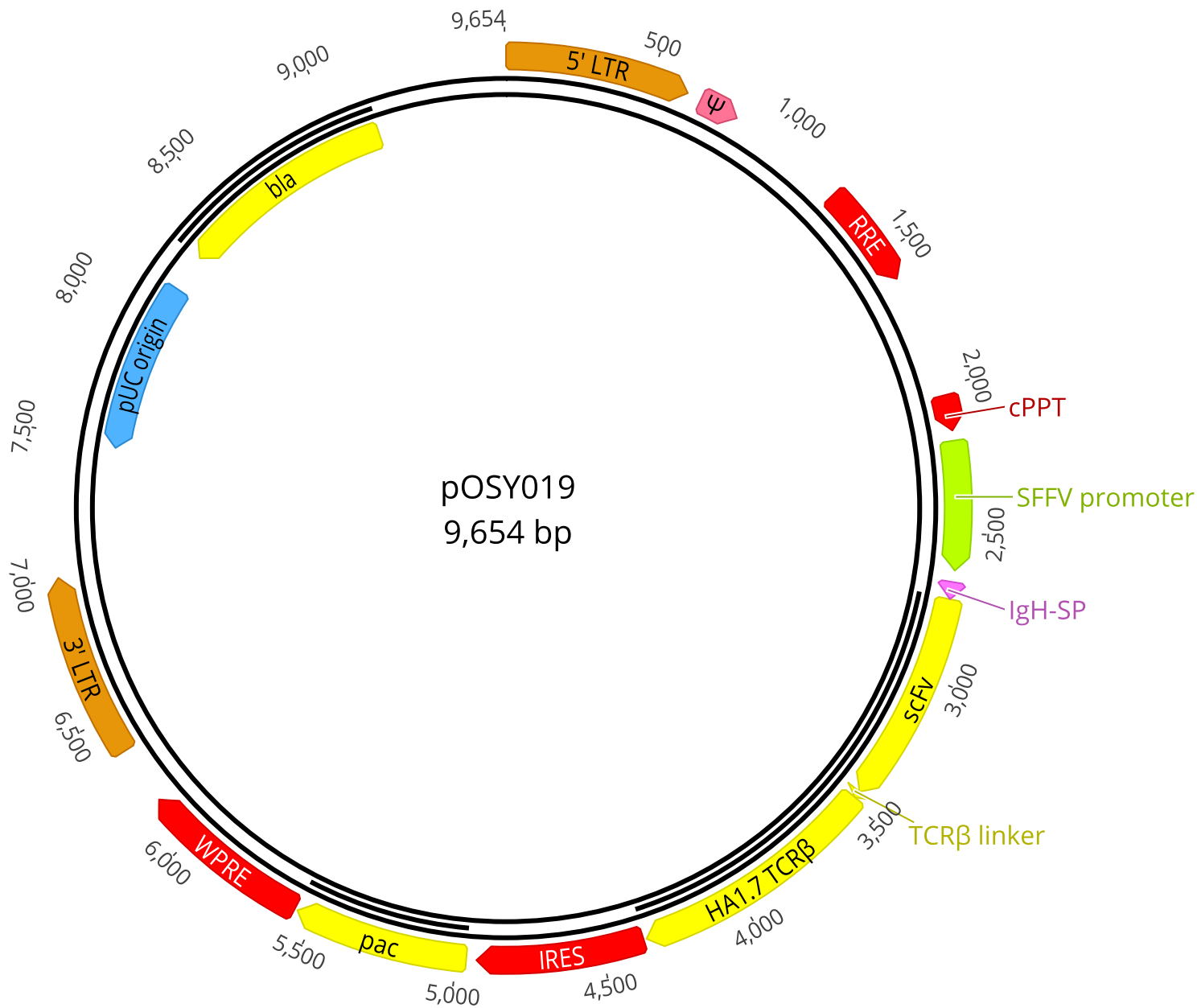

Supplement: Supplementary file 10. [file elife-42475-supp10.pdf]

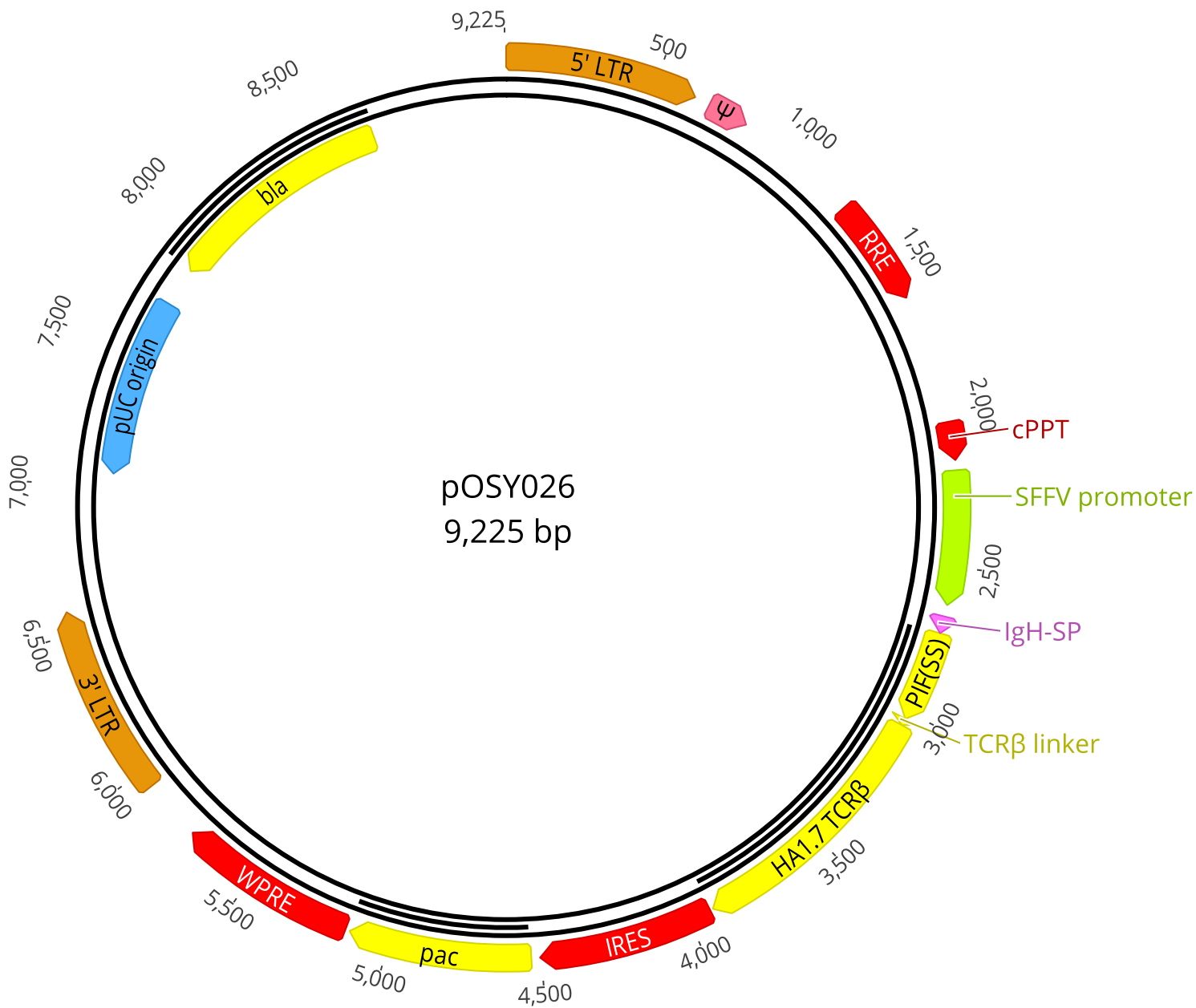

Supplement: Supplementary file 12. [file elife-42475-supp12.pdf]

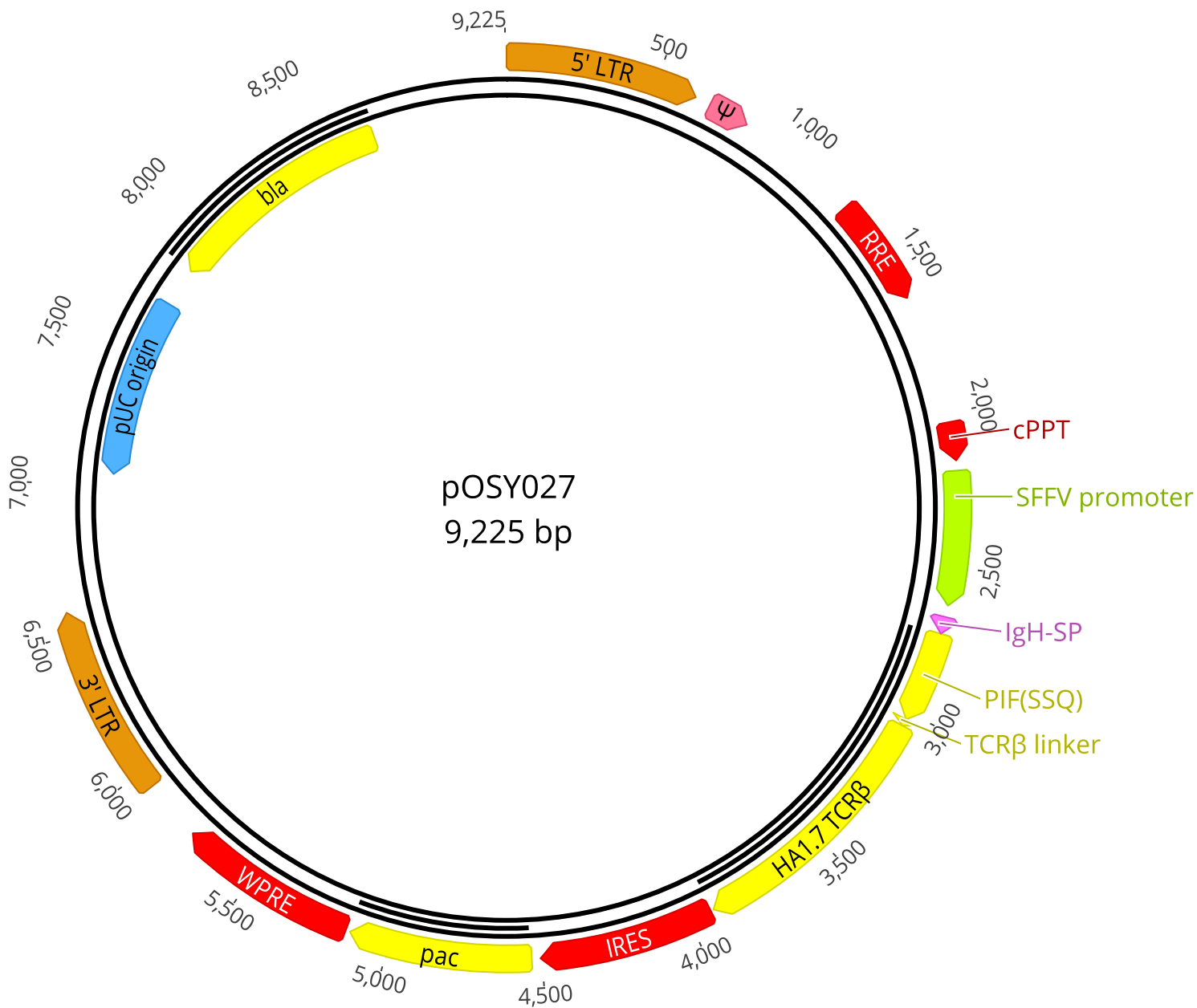

Supplement: Supplementary file 14. [file elife-42475-supp14.pdf]

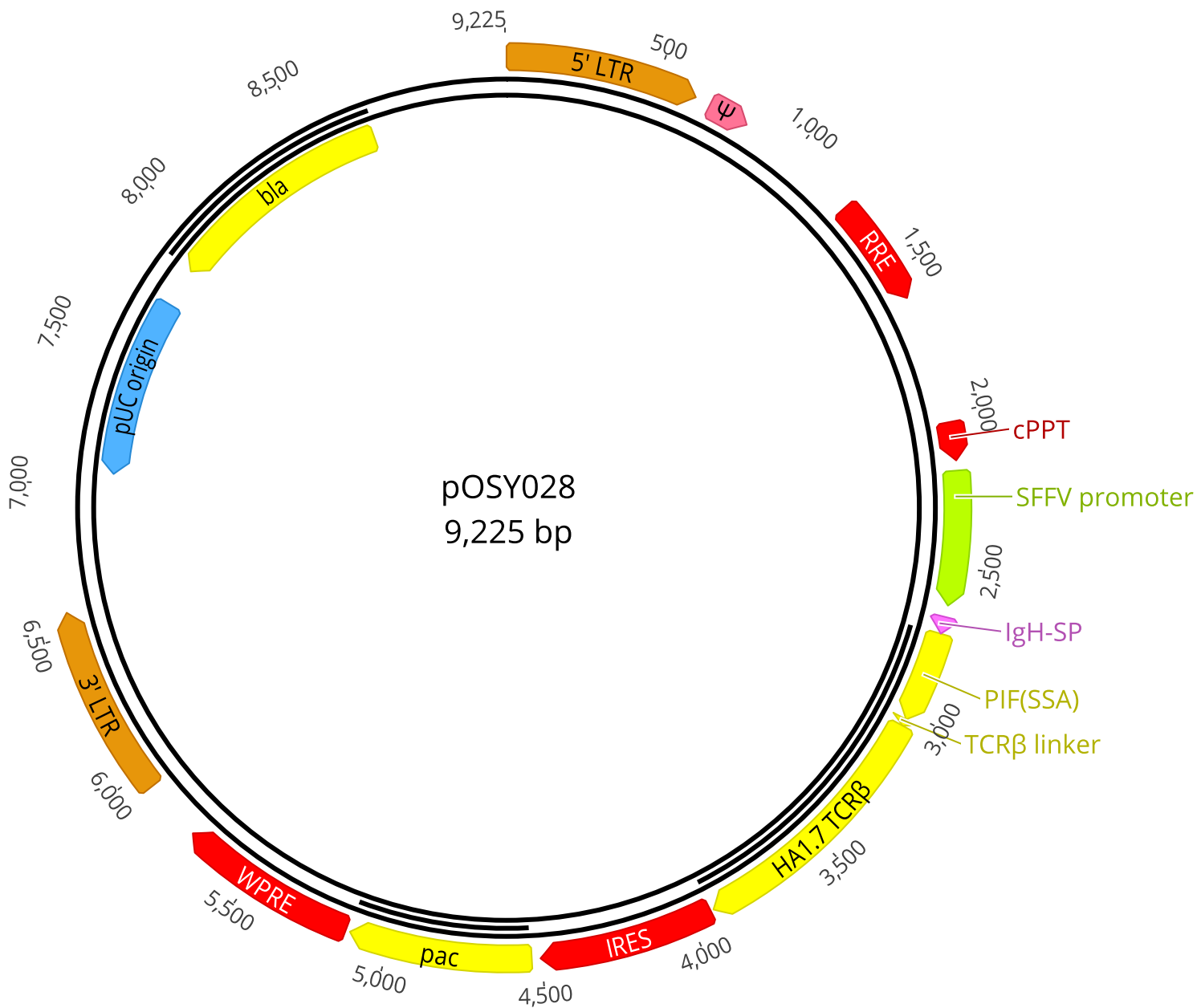

Supplement: Supplementary file 16. [file elife-42475-supp16.pdf]

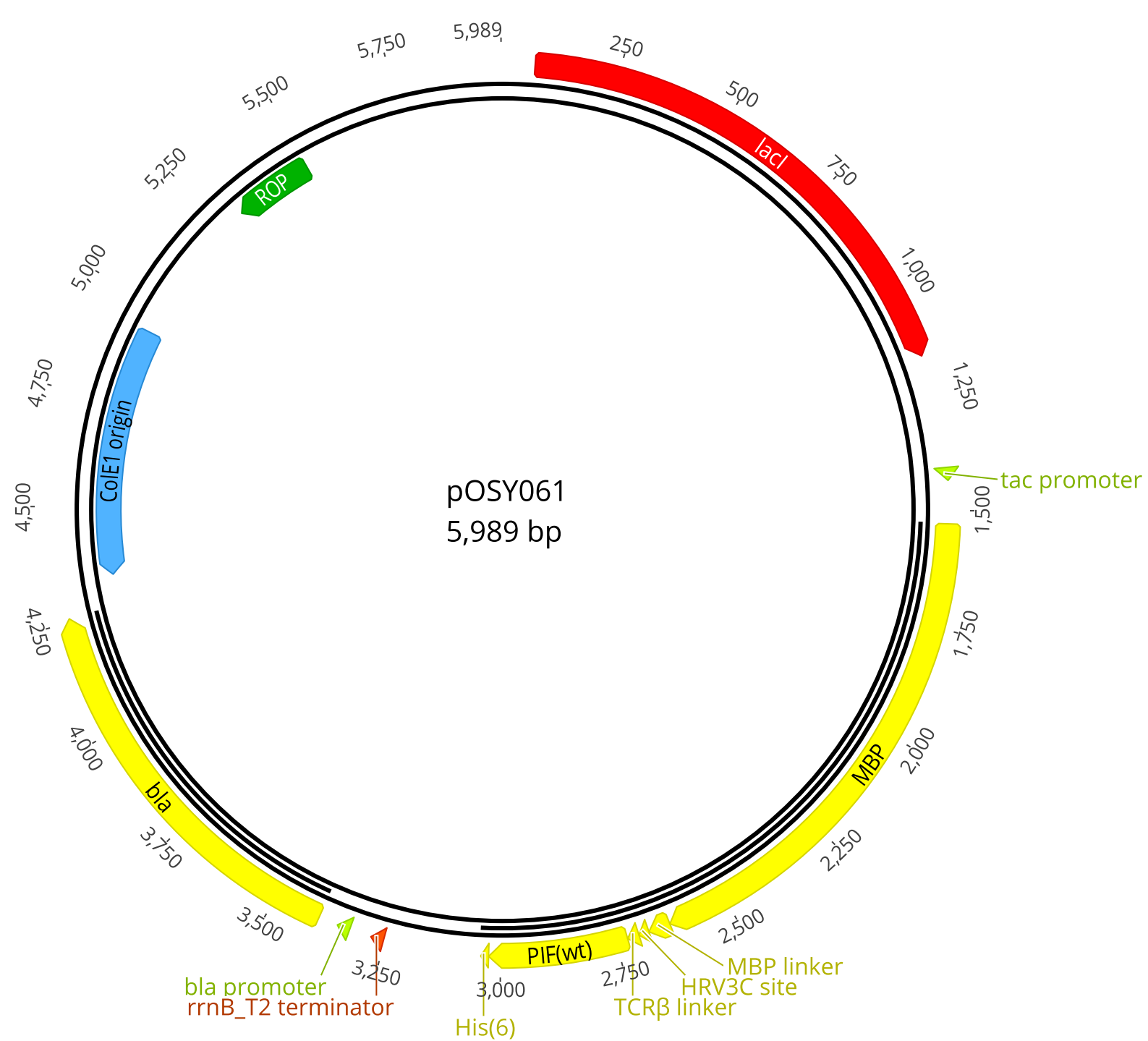

Supplement: Supplementary file 18. [file elife-42475-supp18.pdf]

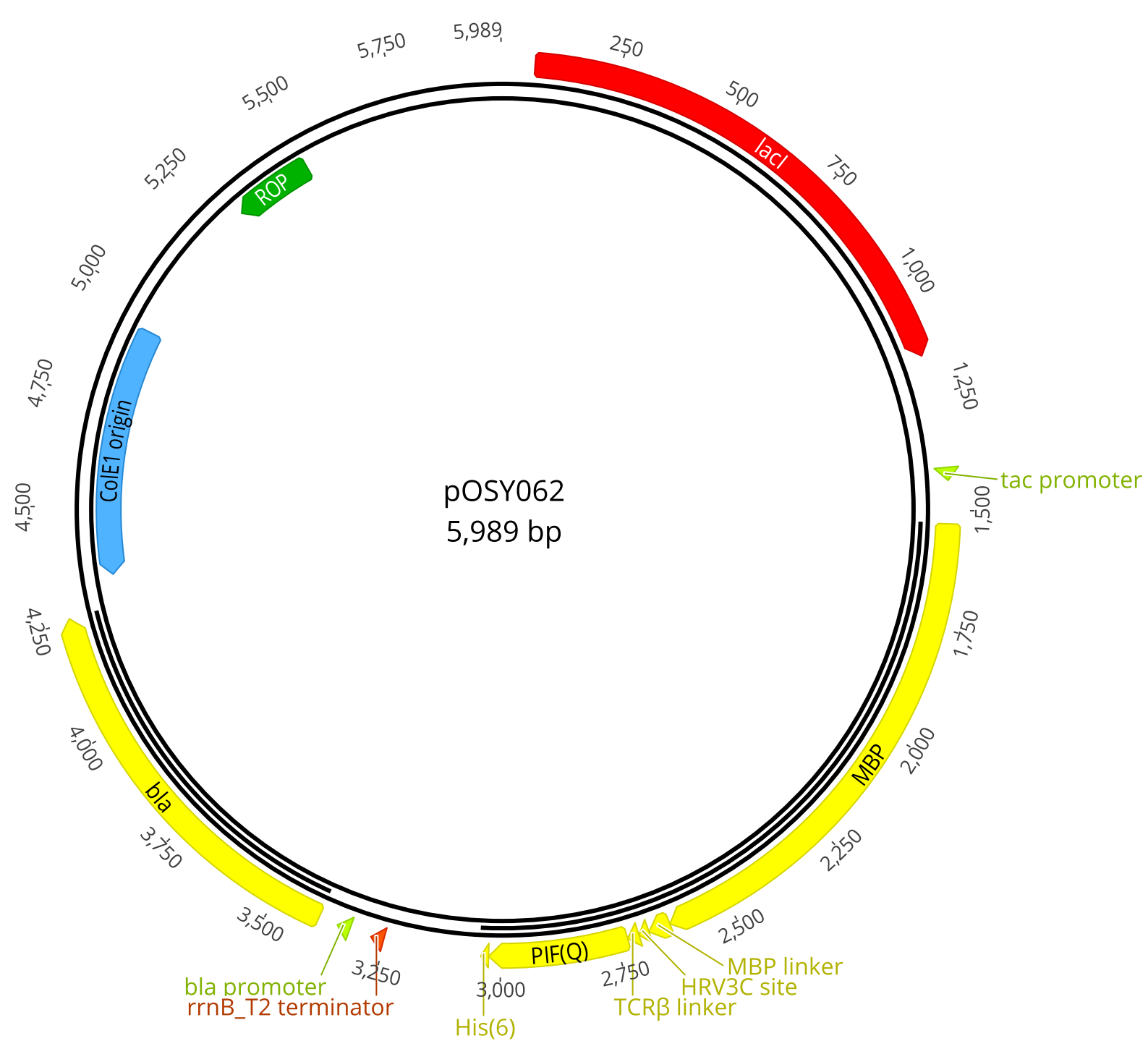

Supplement: Supplementary file 20. [file elife-42475-supp20.pdf]

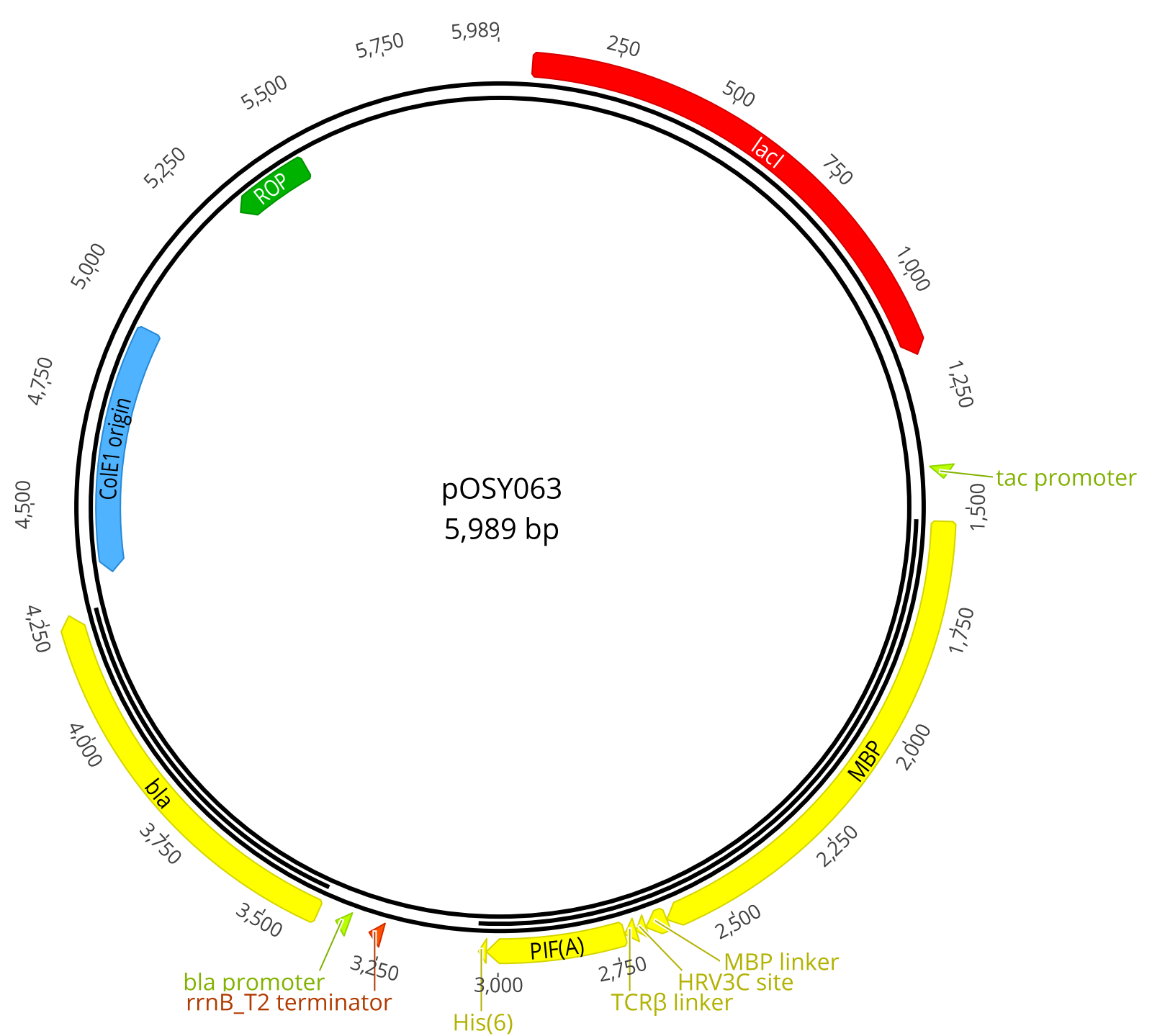

Supplement: Supplementary file 22. [file elife-42475-supp22.pdf]

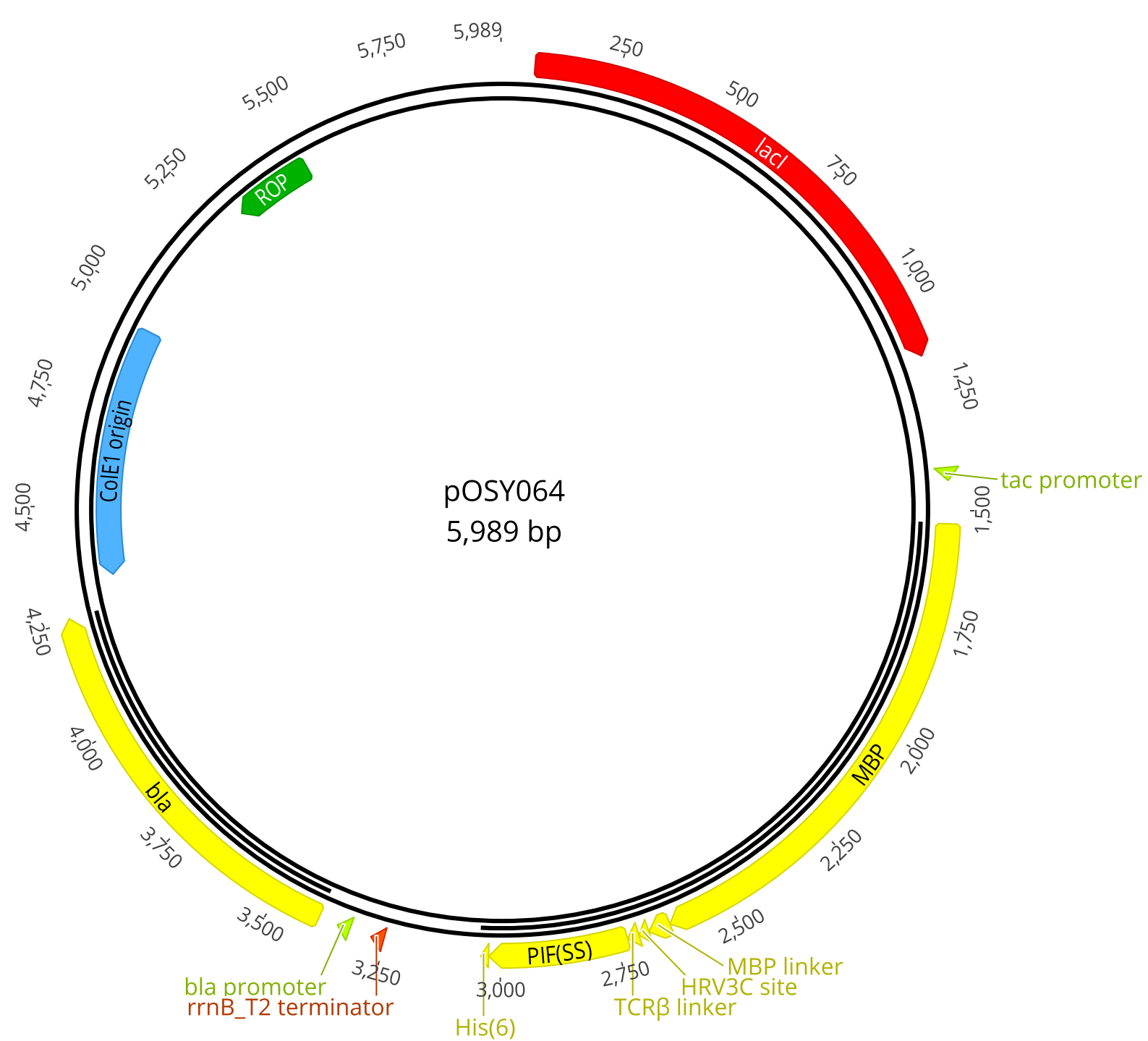

Supplement: Supplementary file 24. [file elife-42475-supp24.pdf]

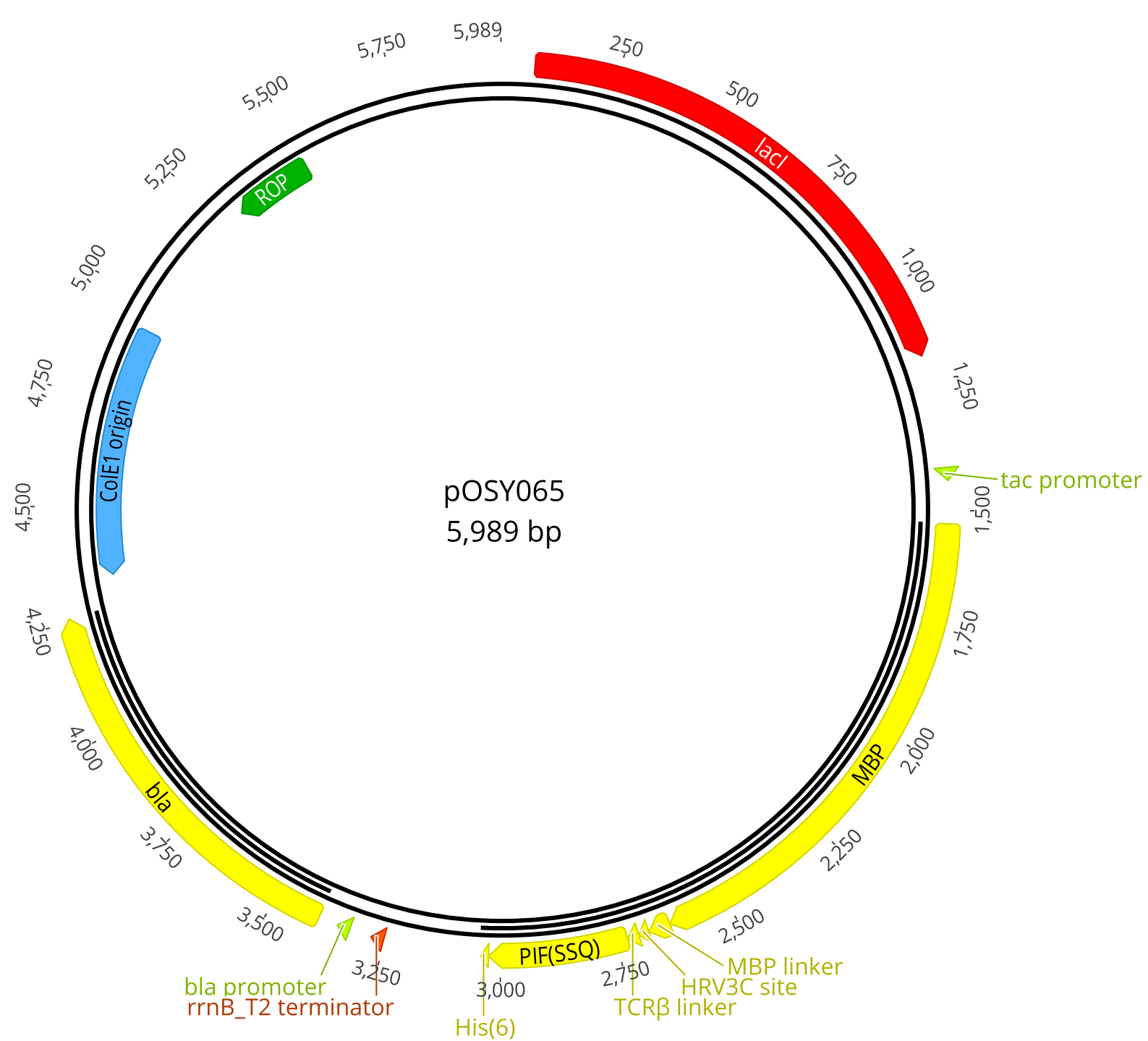

Supplement: Supplementary file 26. [file elife-42475-supp26.pdf]

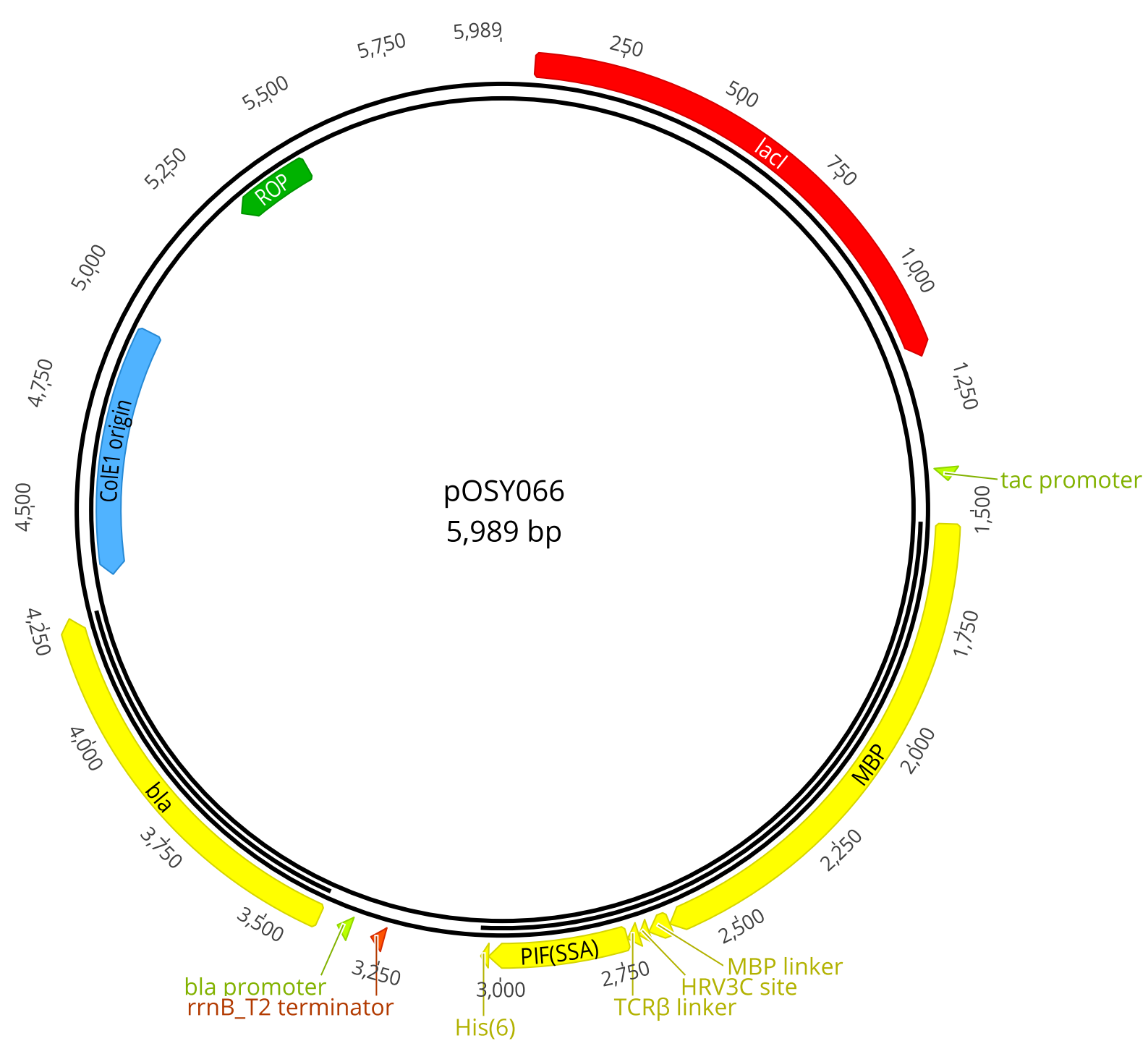

Supplement: Supplementary file 28. [file elife-42475-supp28.pdf]

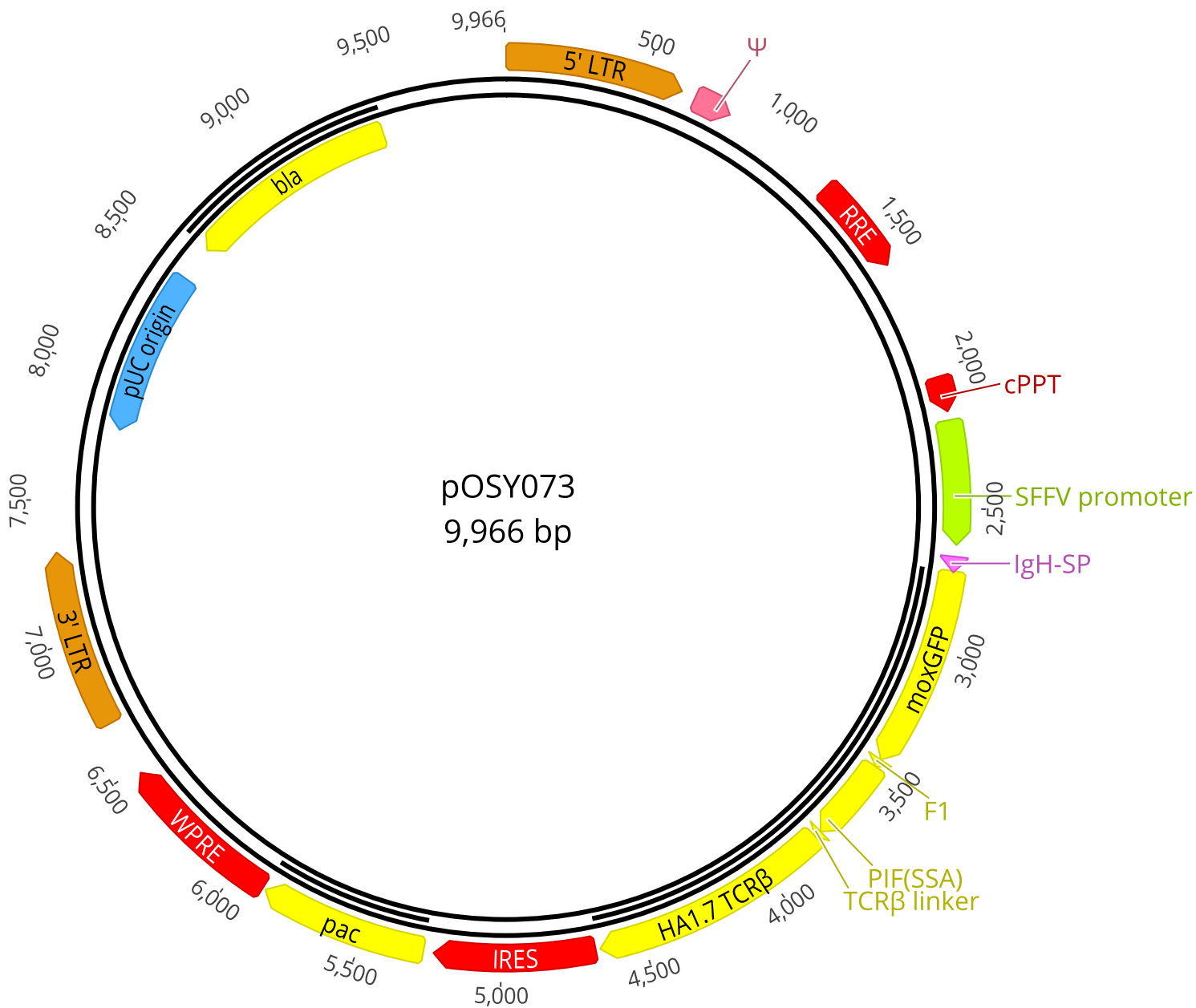

Supplement: Supplementary file 30. [file elife-42475-supp30.pdf]

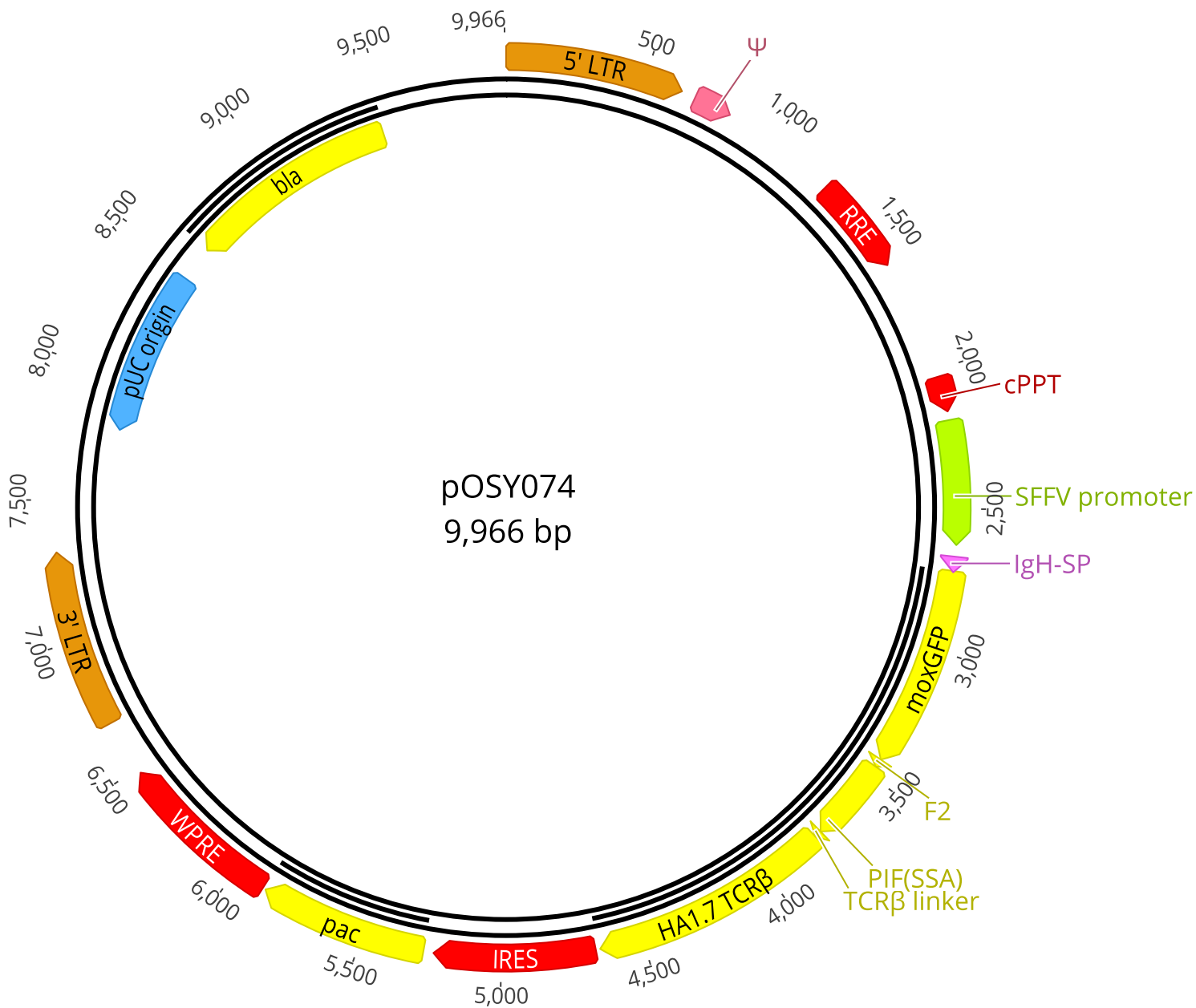

Supplement: Supplementary file 32. [file elife-42475-supp32.pdf]

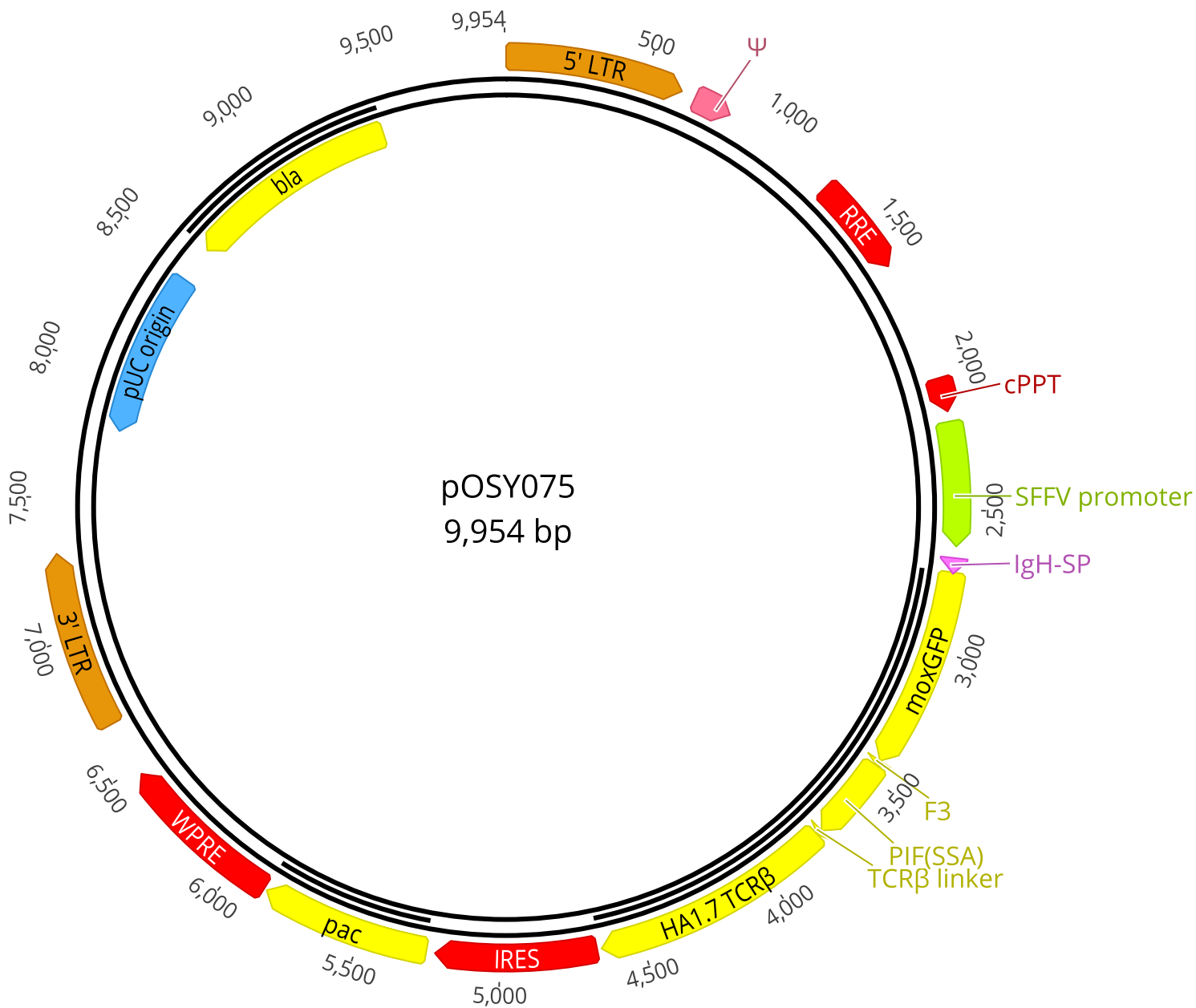

Supplement: Supplementary file 34. [file elife-42475-supp34.pdf]

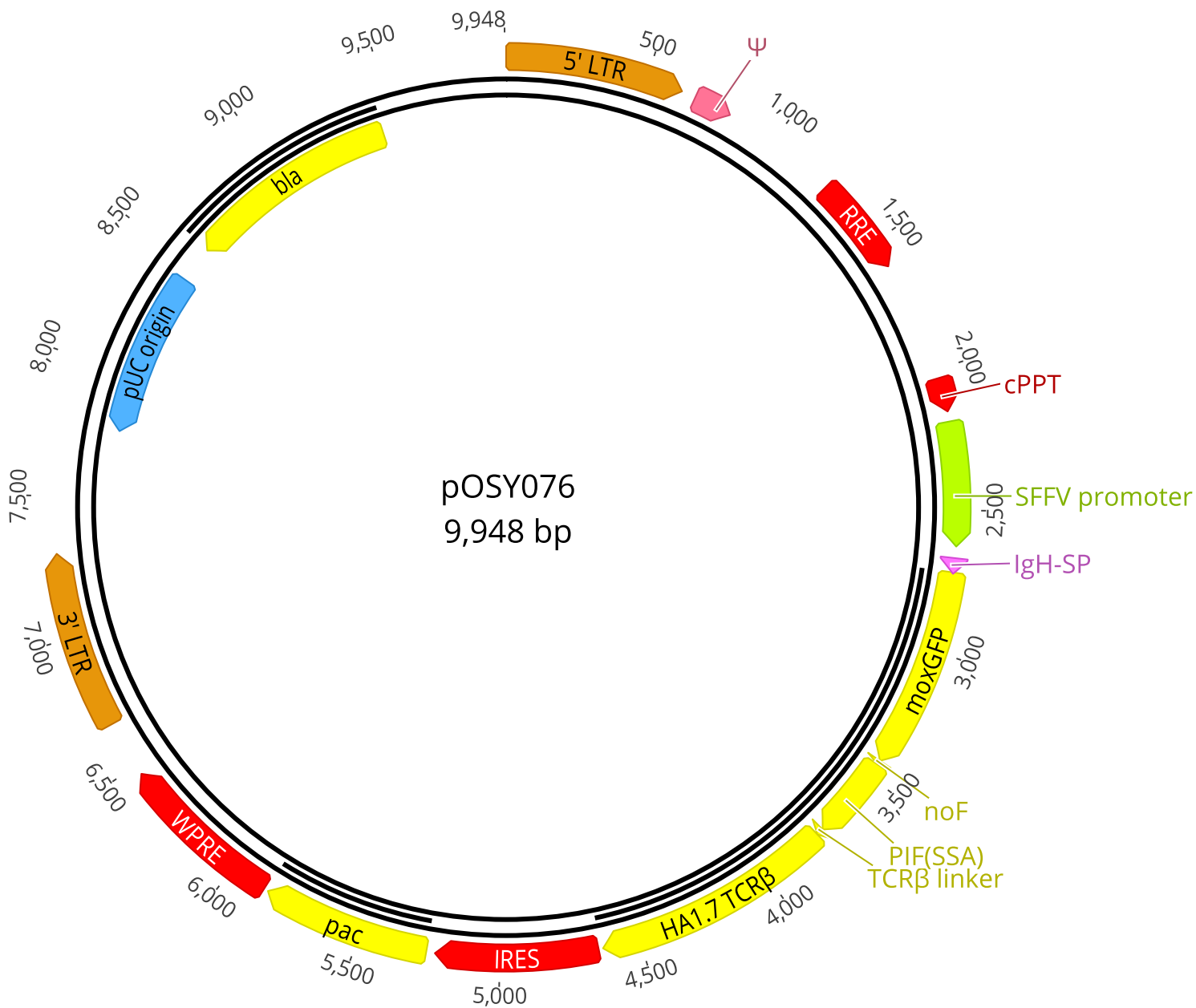

Supplement: Supplementary file 36. [file elife-42475-supp36.pdf]
